# GDF11/Myostatin and aging

**DOI:** 10.18632/aging.100666

**Published:** 2014-05-17

**Authors:** Vishal K. Patel, Fabio Demontis

**Affiliations:** ^1^Medical School, Albert Einstein College of Medicine Bronx, NY 10461, USA; ^2^Department of Developmental Neurobiology Division of Developmental Biology St. Jude Children's Research Hospital Memphis, TN 38105, USA

The aging of a multicellular organism is an intricate process that results from signaling events and age-related degeneration in cells, tissues, and organ systems. Studies in model organisms have provided evidence of unanticipated connection between local and systemic aging [1]. Research in this field is defining the key tissues that govern an organism's lifespan and the signals that convey information about the aging of cells and tissues throughout the organism [1]. Skeletal muscle is now being recognized as a prominent tissue with the capacity to influence systemic aging and lifespan [2, 3]. Many epidemiologic studies in humans have pointed to a remarkable connection between muscle strength and the morbidity and mortality linked to many age-related diseases [2]. The association between muscle function and aging has been corroborated by studies in model organisms. In mice and *Drosophila*, several muscle-restricted genetic interventions induce physiological responses in other tissues, such as regulation of lipid homeostasis in adipose tissue, regulation of insulin release, increased proteostasis, and decreased incidence of many age-related diseases [2, 3]. Although some of these systemic effects may be due to indirect effects of muscles' metabolic demand [2], there is also growing evidence for an endocrine role for skeletal muscle. In particular, muscle-derived cytokines and growth factors known as myokines are becoming recognized as important systemic regulators of metabolic homeostasis and may signal to many target tissues [2].

Using transgenic RNAi screening, we recently discovered several myokines that regulate lifespan and muscle aging in the fruit fly *Drosophila melanogaster* [4]. Among the myokines regulating the lifespan of *Drosophila*, we found Myoglianin, a TGF-beta ligand expressed primarily by skeletal muscle and glia [4]. *Myoglianin* RNAi in muscle shortens lifespan and increases the percentage of age-related climbing defects in the flies while its overexpression has converse effects. *Myoglianin* expression is increased by the transcription factor Mnt in muscle, and Mnt extends lifespan [4]. Modulation of *Mnt* and *myoglianin* expression levels in muscle leads to a partial decrease in rRNA levels and nucleolar function in muscle, a response that itself is sufficient to extend lifespan and that is known to be induced by many interventions that delay aging [4]. In addition to autocrine effects, *myoglianin* overexpression in muscle non-autonomously decreases the size and function of the nucleolus in *Drosophila* adipocytes by activating p38 MAPK [4], a known transducer of non-canonical TGF-beta signaling. Together these findings indicate that muscle-derived Myoglianin systemically regulates nucleolar function across aging tissues in *Drosophila* and that this inter-tissue communication extends lifespan [4].

*Drosophila* Myoglianin is homologous to human GDF11 and Myostatin (GDF8), two highly related TGF-beta ligands that circulate in the bloodstream in mammals [5, 6]. Myostatin is a negative regulator of muscle mass. In addition, it can also signal to non-muscle tissues and, for example, lead to the differentiation of immature adipocytes that protect from obesity and metabolic diseases [6]. We found that *Drosophila* Myostatin (Myoglianin) extends lifespan and delays systemic aging by acting on muscle, adipocytes, and possibly other tissues [4]. These effects were not due to feeding or changes in muscle mass [4], suggesting that *Drosophila* may be a convenient system for testing the direct signaling roles of Myostatin without the indirect confounding effects deriving from the increased muscle mass observed in Myostatin knock-out mice [6]. In fact, these mice have increased insulin sensitivity and decreased adiposity due to higher nutrient utilization in muscle (and consequent reduced nutrient availability for other tissues) deriving from the doubling in muscle mass, which is a prominent feature of Myostatin knock-out mice [6].

In addition to Myostatin, Myoglianin is homologous to the related factor GDF11 [4]. A previous study in mice showed that GDF11 levels decline during aging and that this contributes to developing age-related cardiac hypertrophy [5]. The finding that the GDF11 homolog Myoglianin preserves muscle function during aging in *Drosophila* [4] suggests that GDF11 may also have anti-aging effects on tissues other than the heart. Indeed very recent studies have shown that GDF11 delays skeletal muscle and brain aging in mice [7, 8], suggesting that GDF11 is an evolutionarily conserved, general regulator of tissue aging. The anti-aging responses induced by GDF11 in mice may rely on the nucleolus and p38 MAPK, which is known to delay tissue aging and extend the lifespan of multiple organisms and which is activated by Myostatin in mammals and by Myoglianin in *Drosophila* [4]. Considering that the nucleolus is the site of ribosome biogenesis and its function is necessary for cell growth, GDF11 and Myostatin may prevent heart hypertrophy and perhaps the aging of other tissues by activating p38 MAPK and by decreasing the size and function of the nucleolus. Moderate reductions in nucleolar function, protein synthesis, and cellular anabolism are typical responses induced in mammals by dietary restriction and the lifespan-extending drug rapamycin, and it is therefore possible that GDF11 and Myostatin may in part mimic those treatments. Because excessive decreases in protein anabolism and protein synthesis lead to a loss of muscle mass in mammals, it will be important to fine-tune these interventions to have anti-aging effects without causing sarcopenia and degeneration of other tissues. In mice, Myostatin is expressed primarily in skeletal muscle while *GDF11* has broader tissue expression [5, 6]. Although the spleen has the greatest *GDF11* expression [5], skeletal muscle is the most abundant tissue in an organism, accounting for approximately 40-50% of body mass. Thus, it will be important to learn what is the quantitatively most relevant tissue source of circulating GDF11 in mice and whether *GDF11* expression in muscle can be regulated by the transcription factor Mnt (as observed for *myoglianin* in *Drosophila* [4]) or by other interventions known to affect myokine production, such as physical exercise and nutrient sensing [2].
